# Case Report: ITP Treatment After CAR-T Cell Therapy in Patients With Multiple Myeloma

**DOI:** 10.3389/fimmu.2022.898341

**Published:** 2022-06-16

**Authors:** Mengyi Du, Linlin Huang, Haiming Kou, Chenggong Li, Yu Hu, Heng Mei

**Affiliations:** Institute of Hematology, Union Hospital, Tongji Medical College, Huazhong University of Science and Technology, Wuhan, China

**Keywords:** thrombocytopenia, ITP, CAR-T therapy, multiple myeloma, MAIPA

## Abstract

Chimeric antigen receptor T (CAR-T) cell therapy is an attractive strategy for patients with relapsed or refractory hematological malignancies including multiple myeloma (MM). T cells are engineered to attack malignant cells that express tumor-associated antigens and better efficacy could be achieved. However, cytokine release syndrome (CRS), immune effector cell-associated neurotoxicity syndrome (ICANS), and hematologic toxicity are still challenges for CAR-T cell therapy. Among them, hematologic toxicity including thrombocytopenia has a longer duration and lasting effect during and after the treatment for some patients. Here, we present 3 cases of hematologic toxicity manifested as refractory thrombocytopenia with platelet autoantibodies positive and plasma thrombopoietin (TPO) concentration elevated after bispecific CAR-T cell therapy in relapsed/refractory (R/R) MM patients who were successfully treated with standard therapy of immune thrombocytopenia (ITP). Without clear pathogenesis or guidance on therapy published, our cases provide a reference for the treatment of thrombocytopenia after CAR-T cell therapy and inspire exploration of the underlying pathophysiological mechanisms.

## Introduction

Chimeric antigen receptor T (CAR-T) cells have emerged as a promising treatment approach for hematologic malignancies. Three CAR-T cell products (Kymriah, Yescarta, and Tecartus) have been approved by the US Food and Drug Administration (FDA) thus far (1, 2), and are currently evaluated in patients to enhance efficacy and prolong survival time (3). B-cell maturation antigen (BCMA; also known as TNFRSF17 or CD269) belonging to the tumor necrosis factor (TNF) family, highly expressed on malignant plasma cells, represents an ideal therapeutic target for CAR-T cell therapy in multiple myeloma (MM) (4). The safety and clinical efficacy of BCMA CAR-T cells have been observed (5), and other promising targets including CD38 and CS1 (6) have led to the research on bispecific CAR-T cells to improve the outcome of MM patients.

Despite showing expected therapeutic effects, CAR-T cell therapy may cause adverse effects, such as cytokine release syndrome (CRS) and immune effector cell-associated neurotoxicity syndrome (ICANS), which are of special concern (7, 8). Moreover, hematologic toxicities represented by thrombocytopenia in CAR-T therapy have attracted the attention of clinicians recently (9). Characterized by isolated thrombocytopenia, immune thrombocytopenia (ITP) has been reported in a relapsed/refractory(R/R) MM patient after CAR-T cell therapy (10). The pathogenesis of ITP lies in increased platelet (PLT) destruction and decreased PLT production resulting from autoimmune disorder (11). Autoantibodies targeting PLT glycoproteins (GP) play a major part in PLT destruction, which can be detected by the direct monoclonal antibody immobilization of PLT antigen assay (MAIPA) to assist in diagnosis (12). Here, we review 3 cases of successfully treated thrombocytopenia after CAR-T cell therapy in R/R MM patients with PLT autoantibodies detected by MAIPA, to explore the reason further and provide clinical experience for the treatment of thrombocytopenia after CAR-T therapy.

## Case Presentations

### Case 1

**Figure 1:**
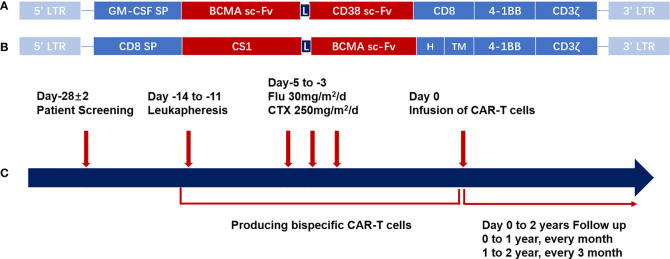
CAR structure and schema of the CAR-T cell therapy. **(A)** Schematic structure of bispecific BM38 CAR. The bispecific BM38 CAR contains an anti-BCMA scFv and an anti-CD38 scFv in tandem in 4-1BB-containing second-generation formats. **(B)** Schematic structure of BCMA/CS1 bispecific CAR. The BCMA/CS1 bispecific CAR contains an anti-CS1 scFv and an anti-BCMA scFv in 4-1BB-containing second-generation formats. **(C)** Diagrammatic sketch of CAR-T cell therapy. After screening for eligibility, PBMCs were collected by leukapheresis for the production of bispecific CAR-T cells, and then patients receive lymphodepleting regimens consisting of CTX (250 mg/m^2^/day, days −5 to −3) and FLU (25 mg/m^2^/day, days −5 to −3). Regular follow-up should be continued after the infusion of CAR-T cells. CAR, chimeric antigen receptor; CAR-T, chimeric antigen receptor-modified T cell; BCMA, B-cell maturation antigen; scFv, single-chain variable region; PBMC, peripheral blood mononuclear cell; CTX, cyclophosphamide; FLU, fludarabine.

A 49-year-old Asian woman was diagnosed with stage I, IgA kappa-type MM according to the revised International Staging System (R-ISS) in 2018 after presenting with low back pain and anemia (13). Then, she received treatment with six chemotherapy cycles, which were poorly tolerated with pneumonia and deep venous thrombosis and achieved no remission (**Table 1**). The patient pinned her hope on CAR-T cell therapy and consented to participate in the clinical trial of bispecific BM38 CAR-T cells targeting BCMA and CD38 (ChiCTR1800018143) (**Figure 1A**). Autologous peripheral blood mononuclear cells (PBMCs) were collected for the generation of CAR-T cells, and after lymphodepleting chemotherapy with 250 mg/m^2^ cyclophosphamide and 30 mg/m^2^ fludarabine for 3 days, CAR-T cells were infused at a dose of 4 × 10^6^ cells/kg (**Figure 1C**).

**Table 1:** Clinical information of 3 patients.

patient		1	2	3
Sex/Age		F/49	M/72	M/72
BMI		20.00	19.03	19.10
Stage		I(R-ISS)	II(R-ISS)	II(D-S)
Classification		IgA-KAP	light chain-KAP	IgD-LAM
Karyotype		del13q14	t(11;14) (q13;32)	normal
Chemotherapy		PAD*3	TD*1	VRCD*10
		VRD*2	BD*19	Lenalidomide
		VAD*1	RD*1	VTD-PACE*2
			Ixazomib*1	
Clinical trial		ChiCTR1800018143	NCT04662099	ChiCTR1800018143
Inclusion reason		refractory	refractory	relapse
CAR structure		BCMA&CD38	BCMA&CS1	BCMA&CD38
Infusion does		4*10^6/kg	0.75*10^6/kg	4*10^6/kg
Lymphodepletion		FLU: 30mg/m2*3d; CTX: 250mg/m2*3d		
CRS (ASTCT)		1	3	1
Efficacy		sCR	VGPR	PR
TCP	occurrence time	15 months afterinfusion	1 month after infusion	2 weeks after infusion
	duration	3m	1.5m	2m
	MAIPA (+)	GPIIB/GPIIIA	GPIIIA	GPIX/GP140
	serum TPO level	397.2 pg/mL	1280pg/mL	2902pg/mL
	treatment	platelet transfusion		
		IVIG and IL-11	rhTPO	IVIG
		rhTPO andeltrombopag	avatrombopag	herombopag
			rituximab	rituximab

F, female; M, male; PAD, (bortezomib, doxorubicin, and dexamethasone); VRD, (bortezomib, lenalidomide, and dexamethasone); VAD, (vincristine, doxorubicin, and dexamethasone); TD, (thalidomide and dexamethasone); BD, (bortezomib and dexamethasone); RD, (lenalidomide and dexamethasone); VRCD, (bortezomib, lenalidomide, cyclophosphamide, dexamethasone); VTD-PACE, (bortezomib, dexamethasone, thalidomide, cisplatin, doxorubicin, cyclophosphamide, and etoposide); FLU, fludarabine; CTX, cyclophosphamide; CRS, cytokine release syndrome; ASTCT, American Society for Transplantation and Cellular Therapy; sCR, stringent complete response; VGPR, very good partial response; PR, partial response; MAIPA, monoclonal antibody immobilization of platelet antigens assay; TCP, thrombocytopenia; TPO,thrombopoietin; IVIG, intravenous immunoglobulin.

**Figure 2:**
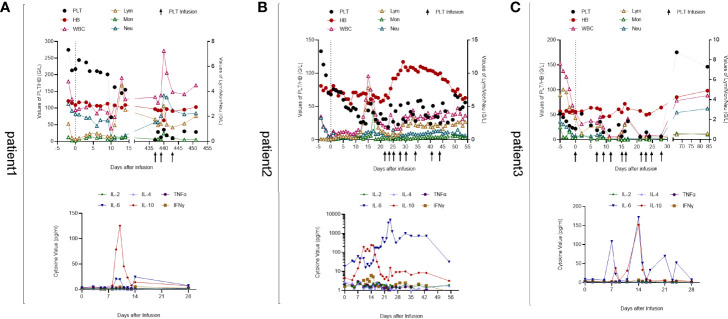
Peripheral blood cell counts and cytokine levels of 3 patients. **(A)** Patient 1. Early cytopenia occurred on day 10 and recovered soon while isolated thrombocytopenia developed later on day 437. The IL-10 level peaked on day 10 and the IL-6 level fluctuated in accordance with IL-10. **(B)** Patient 2. Pancytopenia developed after CAR-T cell infusion. His hemoglobin and leukocyte returned to normal levels 1 month later but thrombocytopenia remained with platelet transfusion necessary. His cytokine levels were significantly higher than those of the other two patients, which may be associated with his gastrointestinal bleeding and cardiovascular disorders. **(C)** Patient 3. His WBC count declined after lymphodepletion chemotherapy. Severe leukopenia and thrombocytopenia occurred 2 weeks after CAR-T cell infusion with blood transfusion inefficacious. At follow-up on day 67, his PLT and WBC returned to normal levels with hemoglobin elevated obviously. IL-6 and IL-10 levels were unstable and fluctuated along with each other.

The PLT count was maintained in the physiological range until CAR-T cell infusion and no immediate severe toxicities occurred on the day of infusion. The patient developed fever within the first week of the treatment and recovered 5 days later without tocilizumab or dexamethasone treatment, which was diagnosed as Grade 1 CRS (14). On day 10 after infusion, the PLT count was reduced to less than half of the prior day (from 201 × 10^9^/L to 72 × 10^9^/L), accompanied by increased interleukin (IL)-6 and IL-10 concentrations in the serum. However, the PLT count returned to normal level the following day with the concentration of cytokines decreased notably (**Figure 2A**). On day 15, there were no plasma cells in the marrow and no M protein detected in the peripheral blood. The serum and urine levels of the kappa chain also decreased to the physiological range. The patient achieved complete response (CR).

**Figure 3:**
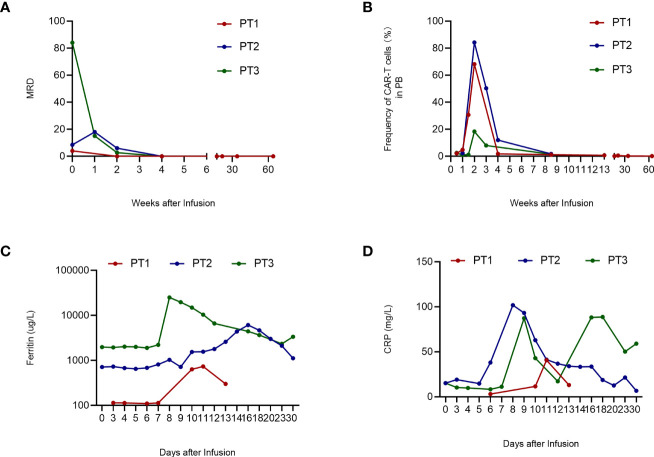
Clinical evaluation after CAR-T cell infusion. **A. MRD of 3 patients.** PT1's MRD turned negative on day 14; PT2’s and PT3’s MRD turned negative on day 21 and remained negative during follow-up. **B. Frequency of CAR-T cells in PB.** The frequency of CAR-T cells in PB peaked on day 2 and then declined gradually. **(C)** Ferritin level of 3 patients. The ferritin level of PT1 was stable within 1 week after CAR-T cell infusion and peaked on day 11. PT2 and PT3 have higher ferritin levels that peaked on day 16 and day 8, respectively. **(D)** CRP level of 3 patients. PT1’s CRP level peaked on day 11; PT2’s CRP level peaked on day 8; PT3’s CRP level has two peaks on days 9 and 18, respectively. WBC, white blood cells; CAR-T, chimeric antigen receptor-modified T cell; PT, patient; MRD, minimal residual disease; CRP, C-reactive protein; PB, peripheral blood.

However, extreme thrombocytopenia and severe adverse events occurred 15 months after CAR-T cell infusion. Scattered subcutaneous bleeding points were observed during a routine follow-up examination without severe bleeding or splenomegaly. Her PLT level decreased to 4 × 10^9^/L with 3.94 × 10^9^/L leucocytes and hemoglobin (Hb) of 91 g/L; three consecutive tests showed PLT below 30 × 10^9^/L. The levels of coagulation indexes and biochemical indexes, such as liver and kidney function level, C-reactive protein (CRP), and lactate dehydrogenase (LDH), were within physiological ranges. Bone marrow aspiration showed the absence of blast cells and thrombogenic megakaryocytes, and the minimal residual disease (MRD) of MM remained negative (**Figure 3A**), though there were no CAR-T cells detected in the peripheral blood (**Figure 3B**). Further tests excluded connective tissue diseases (CTDs) and infections. To explore the reason for thrombocytopenia, thrombopoietin (TPO) concentration and PLT autoantibodies were detected. A high level of TPO concentration (397.2 pg/ml) and the positive detection of anti-GPIIBIIIA were revealed. With no response to PLT infusions, immunoglobulin (20 g/day) and recombinant IL-11 (3 mg/day) were administered for 10 days before she got discharged with a PLT count at 28×10^9^/L. One month later, her PLT level decreased again; hence, she received treatment with recombinant human thrombopoietin (rhTPO) (15,000 U/day) and eltrombopag (25 mg/day), which gradually increased the PLT count to 69 × 10^9^/L, and thrombogenic megakaryocytes were detected by bone marrow cytology examination at the 18th month of follow-up.

### Case 2

A 72-year-old man suffering from worsening pain in his right hip and back was diagnosed with light chain kappa-type MM (R-ISS, stageII) (13) in 2016 (**Table 1**). Then, he received routine chemotherapy for years until severe adverse effects were induced by Ixazomib (**Table 1**). Gastrointestinal bleeding and incomplete intestinal obstruction stopped him from chemotherapy but the disease progressed in March 2021. The patient was refractory and decided to receive CAR-T therapy. BCMA/CS1 bispecific CAR-T cell therapy (NCT04662099) (**Figure 1B**) was chosen after screening and then autologous T cells were collected. After lymphodepletion with fludarabine (30 mg/m^2^×3 days) and cyclophosphamide (250 mg/m^2^×3 days), CAR-T cells were infused at a dose of 0.75 × 10^6^ cells/kg twice in consideration of the patient’s cardiovascular disorders.

PLTs remained at normal levels until the end of lymphodepleting chemotherapy. On the day of infusion, the PLT count decreased to 68 × 10^9^/L, and in the second week after that, the patient developed pancytopenia with elevated levels of IL-10 (**Figure 2B**), ferritin (**Figure 3C**), and CRP (**Figure 3D**). Fever occurred on day 10 with hypoxemia and hypertension and grade III CRS was diagnosed (14). On day 15, the patient suffered from gastrointestinal bleeding with significantly increased IL-6 levels (**Figure 2B**). With the assistance of gastroenterologists and the therapy of Tocilizumab and methylprednisolone, the bleeding was successfully stopped. After 1 month of CAR-T cell infusion, hemoglobin and leukocyte count recovered, but severe thrombocytopenia remained (23 × 10^9^/L) despite frequent PLT transfusion and rhTPO injection (**Figure 2B**). Marrow aspirate showed the absence of plasma cells and MRD negativity (**Figure 3A**), though there is still urine M-protein detectable by immunofixation. The patient was defined as very good partial response (VGPR). Since the TPO receptor agonist (TPO-RA) has been reported to be effective in thrombocytopenia after CAR-T therapy (15), the patient received avatrombopag (40 mg, qd) orally for 2 weeks. Disappointedly, there is no response either.

The patient was transferred to the gastroenterology department for further care after tests of plasma TPO concentration and MAIPA. The TPO concentration was 1,280 pg/ml, much higher than the upper limit, which could be the reason why he did not respond to avatrombopag. The result of MAIPA revealed the presence of anti-GPIIIA and inspired us to treat with rituximab. As a consequence, the patient’s PLT count gradually increased after treatment and remained above 50 × 10^9^/L without PLT transfusion until he was discharged 2 weeks later (**Figure 2B**).

### Case 3

A 72-year-old man was diagnosed as IgD lambda-type MM (D-S, stage II group A) (16) in 2019 (43% bone marrow naïve plasma cells). Then, he received 9 cycles of chemotherapy until CR, which was followed by maintenance therapy with lenalidomide and regular follow-up (**Table 1**). Tests revealed disease relapse 1 year later and chemotherapy achieved no satisfactory efficacy. CAR-T therapy became the last choice. The patient took part in the clinical trial of bispecific BM38 CAR-T cells (ChiCTR1800018143) (**Figure 1A**) and was infused with 4 × 10^6^/kg of autologous T cells expressing a bispecific CAR targeting BCMA and CD38 after apheresis and lymphodepletion with fludarabine (30 mg/m^2^ × 3 days) and cyclophosphamide (250 mg/m^2^ × 3 days) (**Figure 1C**).

The patient had severe anemia and thrombocytopenia but normal leukocyte counts before the treatment, and blood transfusions were necessary. No obvious change appeared in the hemogram until 2 weeks after CAR-T cell infusion through PLT transfusion was still given frequently. However, the PLT count decreased to even lower on day 15, with severe leukopenia and elevated levels of IL-6 and IL-10 (**Figure 2C**). There were only 7 × 10^9^/L PLTs and remained below 20 × 10^9^/L after PLT transfusion (**Figure 2C**). Cross-match PLT transfusion was inefficacious either. Bone marrow aspirate showed no plasma cells and the MRD was negative on day 21 (**Figure 3A**). Partial response (PR) was defined according to the serum M protein. Since the patient had decreased gamma globulin and severe thrombocytopenia, intravenous immunoglobulin (IVIG) was administered for three consecutive days, and then herombopag was taken orally at 2.0 mg/day until he got discharged. Furthermore, plasma TPO concentration and MAIPA were tested to confirm the reason for thrombocytopenia and guide treatment before discharge. Results showed significantly elevated TPO concentration (2,902 pg/ml) and positive for anti-GPIX and anti-GMP140. Thus, he was then treated with rituximab (100 mg/week for 4 weeks) in a local hospital as our direction. At follow-up on day 67, the patient’s PLT and leukocytes returned to normal levels with hemoglobin elevated notably (**Figure 2C**).

## Discussion

### Incidence of Post-CAR-T Thrombocytopenia

CAR-T cell therapies harness the power of the immune system to recognize and eliminate cancer cells and have shown promising clinical efficacy in hematologic malignancies, providing high response rates, long remission, and improved survival (17, 18). However, adverse effects such as CRS, ICANS, and hematologic toxicity call for special concerns (7, 8). In a long-term follow-up study of patients treated with CD19-targeted CAR-T cells, 16% of them experienced prolonged cytopenia requiring transfusions or growth factor support with ongoing CR and with no MDS (9). It is also reported that persistent CTCAE grade ≥3 thrombocytopenia occurs at a high incidence (21%–29%) (19). Expected to be related to lymphodepleting chemotherapy, early thrombocytopenia typically occurs within a median of 0 days (range, 0–38 days) after CAR-T cell infusion, with a median duration of 32 days (range, 1–64 days), usually accompanied by leukopenia and anemia. Isolated thrombocytopenia appeared more frequently in the late phase and there may be no interim recovery between the two phases (9).

### Case Review and Clinical Suggestions

ITP was previously reported in a patient with relapsed MM after BCMA CAR-T cell therapy and was well treated with rhTPO and immunoglobulin (10). A variety of autoimmune diseases are diagnosed “secondary” to hematologic neoplasms, including autoimmune cytopenia (autoimmune hemolytic anemia, ITP, and Evans syndrome) (20, 21). Therefore, ITP could develop after CAR-T cell therapy and induce severe thrombocytopenia. In the cases we presented above, patient 1 and patient 2 developed isolated thrombocytopenia 15 months and 1 month after CAR-T cell infusion, respectively, without malignant infiltration of the bone marrow and signs of dysplasia typical for MDS, and there was no evidence for other causes of secondary thrombocytopenia either. ITP can be diagnosed and follow-up should be continued. As for patient 3, severe thrombocytopenia and leukopenia developed after CAR-T cell infusion, though it is not clear whether there was isolated thrombocytopenia in a lack of detailed information in a local hospital. Thus, we are not able to confirm the diagnosis of ITP because it could also be cytopenia induced by myelosuppression, but fortunately, they all benefited from the standard therapy of ITP.

Patient 1 had no response to IVIG and IL-11 but the PLT count increased to 69 × 10^9^/L after treatment with rhTPO and eltrombopag. As for patient 2, PLT transfusion and rhTPO could hardly increase the PLT count and avatrombopag was inefficacious. Patient 3 was refractory to PLT transfusion, and subsequent treatment with rhTPO, immunoglobin, and herombopag received no response either. Both patient 2 and patient 3 underwent MAIPA and plasma TPO concentration tests after TPO-RA treatment failed. Significantly increased TPO levels and the existence of PLT autoantibodies inspired us to treat them with rituximab, which took effect at last. Though patient 1 has similar test results to these two patients, she was well treated with rhTPO and eltrombopag. The probable explanation may be that she had relatively lower plasma TPO concentration and anti-GPIIbIIIa achieves a better response to immunoglobin in ITP (22).

Without a specific diagnostic test, direct MAIPA could assist in the diagnosis of ITP and help to choose treatment and monitor the physiological change in clinical practice, which contributes to the personalized treatment of ITP (12). Studies have also shown that elevated TPO levels may predict a lack of response to TPO agonists and the absence of PLT autoantibodies is associated with refractoriness to rituximab (23, 24). As a classic treatment of ITP, glucocorticoid has limitations in patients receiving CAR-T therapy, while immunoglobin and rhTPO become the first choice. TPO-RAs, such as eltrombopag, which are widely used and effective in thrombocytopenia caused by ITP and other diseases, are preferred when traditional treatments failed. With the killing effects on CD20^+^ B cells, rituximab is an optimal solution for patients with PLT autoantibody. Based on experience with the patients above and published cases (15), guidelines for ITP could be referred in post-CAR-T thrombocytopenia, and lab examinations are also necessary to identify the pathogenesis in different patients.

### Possible Pathogenesis of Post-CAR-T Thrombocytopenia

Adverse toxicities in CAR-T therapy are associated with cytokines such as IL-6 and IL-10 (17). IL-6 promotes Th17 differentiation and suppresses regulatory T cells (Tregs) and is also important for B-cell differentiation and plasma cell survival (25). IL-10 is known as a major suppressor of the immune response, but there is recent evidence that it also stimulates the immune response and enhances cytotoxic activity in some contexts (26). Elevated IL-6 and IL-10 levels were observed in ITP patients compared to healthy controls though some researchers claimed that no significant differences in cytokine levels were revealed (27–29). The pathogenesis of ITP after CAR-T therapy remains unclear, but increased levels of IL-6 and IL-10 after CAR-T cell infusion may reflect the immune status of patients: elevated IL-6 and IL-10 impair the balance of Treg/Th17 cells and activate CD8^+^ T cells and antibody production, which led to the immune destruction of PLTs. ITP after anti-BCMA CAR-T therapy in MM has been reported, and there are also cases of lenalidomide-associated ITP (10, 30). With the development of CAR-T therapy, there would be more patients presenting with thrombocytopenia after CAR-T cell infusion. Awareness of ITP after CAR-T therapy should be raised, and ITP and immune-related indicators need to be monitored to explore the underlying mechanisms of post-CAR-T thrombocytopenia.

## Conclusions

CAR-T therapy has shown its efficacy in hematological malignancy. The risk is inevitable when it brings hope of life to patients with R/R disease. Thrombocytopenia is a common problem that occurred in patients after CAR-T therapy, and when isolated thrombocytopenia appears, ITP should be taken into consideration. The underlying pathophysiological mechanisms of ITP after CAR-T therapy remain unexplored, but the standard therapy of ITP is effective for post-CAR-T thrombocytopenia, suggesting at least partial overlap in pathogenesis. However, more clinical cases and further studies are needed to confirm our hypothesis.

## Data Availability Statement

The original contributions presented in the study are included in the article/supplementary material. Further inquiries can be directed to the corresponding authors.

## Ethics Statement

The studies involving human participants were reviewed and approved by the Medical Ethics Committee of the Union Hospital affiliated to Huazhong University of Science and Technology, Wuhan, China. The patients/participants provided their written informed consent to participate in this study. Written informed consent was obtained from the individual(s) for the publication of any potentially identifiable images or data included in this article.

## Author Contributions

MD and LH drafted the manuscript and figures. HK and CL managed the patient. HM and YH were in charge of the final approval of the manuscript. All authors contributed to the article and approved the submitted version.

## Funding

This research was supported by grants from the National Key R&D Program of China (Grant No. 2019YFC1316203), the Natural Science Foundation of Hubei Province (Grant No. 2020CFA065), and the National Natural Science Foundation of China (Grant No. 82070124).

## Conflict of Interest

The authors declare that the research was conducted in the absence of any commercial or financial relationships that could be construed as a potential conflict of interest.

## Publisher’s Note

All claims expressed in this article are solely those of the authors and do not necessarily represent those of their affiliated organizations, or those of the publisher, the editors and the reviewers. Any product that may be evaluated in this article, or claim that may be made by its manufacturer, is not guaranteed or endorsed by the publisher.
